# Primary Small Cell Carcinoma of the Larynx: Report of a Rare Tumor

**DOI:** 10.1155/2011/978676

**Published:** 2011-09-22

**Authors:** Harmeet Singh, Ashok Chauhan

**Affiliations:** Department of Radiotherapy, Post Graduate Institute of Medical Sciences, Rohtak 124001, India

## Abstract

Primary small cell carcinoma of larynx is a rare tumor representing less than 0.5% of all laryngeal cancers. This is one of the most lethal of all malignancies associated with frequent early widespread metastases and dismal prognosis. We report a case of a 60-year-old patient with small cell carcinoma of the subglottic larynx, who presented with hepatic metastasis.

## 1. Introduction

Primary laryngeal carcinomas comprise approximately 2% to 5% of all malignancies worldwide. Of these laryngeal carcinomas, approximately 99% are primary squamous cell carcinomas [1]. First reported in 1972, the primary small cell carcinoma of larynx is a rare tumor representing less than 0.5% of all laryngeal cancers [2]. This is one of the most lethal of malignancies associated with frequent and early widespread metastases and dismal prognosis [3]. During the past 30 years, about 160 cases of primary small cell carcinoma of the larynx have been reported worldwide [1]. Most of the patients are between 50 to 70 years of age [1]. Men are more commonly affected [2]. It has been associated with heavy smoking [2]. Symptoms are similar to those associated with other laryngeal carcinomas. Because of its nonspecific clinical and radiological manifestations, the diagnosis of small cell carcinoma of the larynx is essentially based on the light microscopic examination aided by electron microscopy or immunohistochemical staining [4]. Primary small cell carcinoma of the larynx continues to pose problems in its treatment. Two- and 5-year survival have been reported to be 16 and 5%, respectively [2]. The only variables, which significantly affect survival, are the presence of metastases at initial examination and treatment modality. No association has been found with the stage of the disease [5]. 

## 2. Case Summary

**Figure 1:**
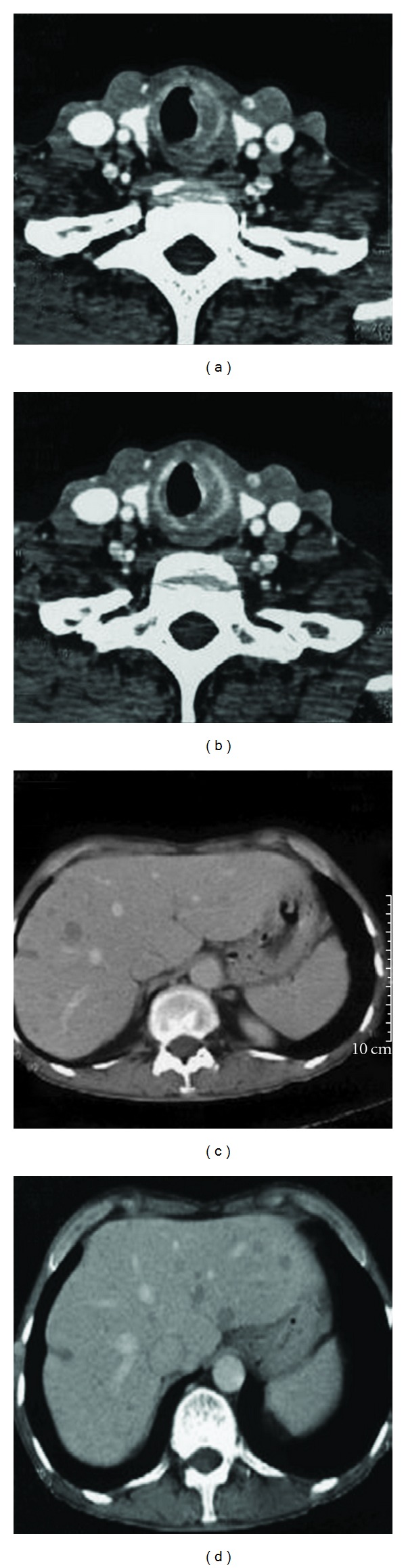
CT scan of the patient showing the primary tumor in the subglottic region.

A 60-year-old male reported with the complaints of throat pain and hoarseness of voice for 3 months. The patient had been smoking 2 packs of cigarette per day for the past 25 years. Direct laryngoscopy revealed a 2 cm growth below the vocal cords. Contrast Enhanced Computed Tomography (CECT) of neck showed growth in subglottic region, and CECT thorax and abdomen showed multiple hypodense lesions in both the lobes of liver suggestive of metastasis (Figure 1). 

**Figure 2:**
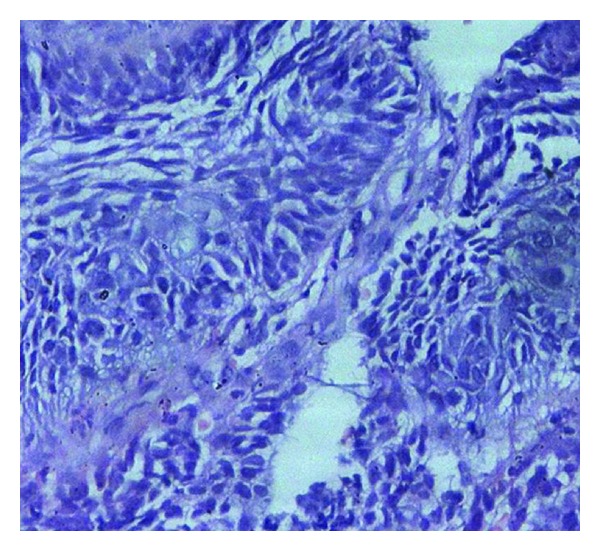
Photomicrograph showing sheets of small round cells with a scant amount of cytoplasm and hyperchromatic nuclei. (H&E; ×200.).

A biopsy was taken from the lesion, and histopathological examination revealed small round cells with scant amount of cytoplasm and hyperchromatic nuclei suggestive of a round cell tumor (Figure 2).

**Figure 3:**
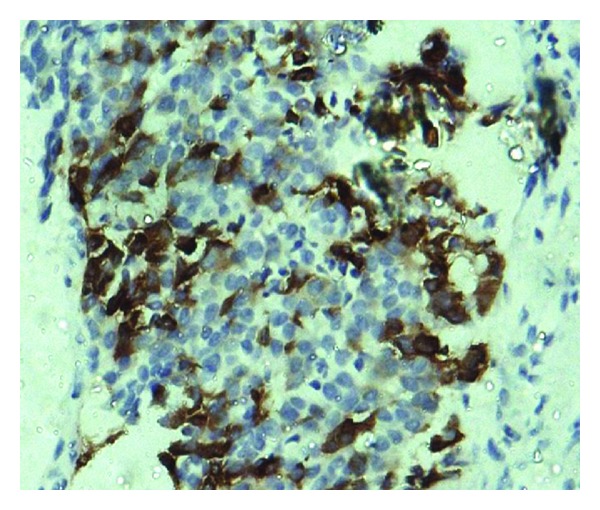
Photomicrograph showing tumor cells positive for synaptophysin.

Immunohistochemistry (IHC) examination showed that the tumor cells were positive for synaptophysin (Figure 3) and cytokeratin. CD-20, CD-3, and LCA were negative. So the diagnosis of small cell carcinoma was made.

The patient was given palliative external radiotherapy in a dose of 20 Gy in five fractions over one week. After the completion of radiotherapy the patient was given combination chemotherapy with cisplatin and etoposide. The general condition of the patient improved initially with therapy and he remained asymptomatic with disease for almost 10 months after which he developed bone pains. A radioisotope bone scan revealed multiple skeletal metastases. The general condition of the patient was too poor to tolerate any chemotherapy. He was offered best supportive care but he succumbed to his disease 12 months after diagnosis.

## 3. Discussion

The WHO has characterized neuroendocrine tumor of the larynx into four types: typical carcinoid tumor, atypical carcinoid tumor, small-cell neuroendocrine tumor, and paraganglioma [6]. An accurate histologic diagnosis is essential because the treatment and prognosis depends on the type of neuroendocrine tumor [6]. Small cell neuroendocrine neoplasm of the larynx has the worst prognosis, with five-year survival rates of 5% [7]. Commonly, cervical and distant metastases are seen at the time of diagnosis [2, 7]. The treatment of small cell carcinoma of the larynx remains controversial as small number of patients has been reported in the literature and there is lack of controlled studies. The aggressive nature of small cell carcinoma of the larynx presents a challenge to the oncologist [8]. Reports of improved survival with systemic chemotherapy combined with radiation therapy had been suggested as a primary treatment modality for laryngeal small cell carcinoma [9]. Cisplatin, etoposide, cyclophosphamide, doxorubicin, vincristine, and methotrexate have been the most commonly used chemotherapeutic agents [7, 9]. Longer survival has been observed in patients who receive chemotherapy [1, 9, 10]. In the present case, the patient was given radiotherapy for palliation of local symptoms followed by systemic chemotherapy with cisplatin and etoposide combination, which improved the general condition of the patient for nearly ten months and the patient survived for a year despite presenting with metastatic disease. Recent reports also suggest that surgery is not indicated, and radiation therapy and systemic chemotherapy appear to be the least disabling and the most efficacious form of treatment for the small cell carcinoma of the larynx [6, 10]. Surgery is best reserved for persistent and recurrent disease at the primary site and neck [9].

## References

[1] Jaiswal VR, Hoang MP (2004). Primary combined squamous and small cell carcinoma of the larynx: a case report and review of the literature. *Archives of Pathology and Laboratory Medicine*.

[2] Gnepp DR (1991). Small cell neuroendocrine carcinoma of the larynx. A critical review of the literature. *Journal for Oto-Rhino-Laryngology and Its Related Specialties*.

[3] Baugh RF, Wolf GT, Beals TF, Krause CJ, Forastiere A (1986). Small cell carcinoma of the larynx: results of therapy. *Laryngoscope*.

[4] Kim HJ, Hwang EG (1997). Small cell carcinoma of the larynx: imaging findings. *Auris Nasus Larynx*.

[5] Soussi AC, Benghiat A, Holgate CS, Majumdar B (1990). Neuro-endocrine tumours of the head and neck. *Journal of Laryngology and Otology*.

[6] Kayhan FT, Basaran EG (2010). Typical carcinoid tumor of the larynx in a woman: a case report. *Journal of Medical Case Reports*.

[7] Ferlito A, Silver CE, Bradford CR, Rinaldo A (2009). Neuroendocrine neoplasms of the larynx: an overview. *Head and Neck*.

[8] Giddings NA, Kennedy TL, Vrabec DP (1987). Primary small cell carcinoma of the larynx: analysis of treatment. *Journal of Otolaryngology*.

[9] Monroe AT, Morris CG, Lee E, Mendenhall WM (2005). Small cell carcinoma of the head and neck: The University of Florida experience. *Journal of the Hong Kong College of Radiologists*.

[10] Sole J, Jurgens A, Musulen E (1994). Small cell carcinoma of the larynx: results of therapy. *Bulletin du Cancer/Radiotherapie*.

